# Rate control in atrial fibrillation using Landiolol is safe in critically ill Covid-19 patients

**DOI:** 10.1186/s13054-021-03470-3

**Published:** 2021-01-22

**Authors:** Geoffroy Hariri, Tomas Urbina, Sandie Mazerand, Naike Bige, Jean-Luc Baudel, Hafid Ait-Oufella

**Affiliations:** 1 Service de réanimation médicale, Assistance Publique – Hôpitaux de Paris (AP-HP), Hôpital Saint-Antoine, 184 Rue du Faubourg Saint-Antoine, 75571 Paris Cedex 12, France; 2 Sorbonne Université, Paris, France; 3 Inserm U970, Centre de Recherche Cardiovasculaire de Paris (PARCC), Paris, France

## Dear editor,

Atrial fibrillation (AF) is frequent in shock patients admitted to the intensive care unit (ICU) [1, 2] and is associated with increased mortality [1]. Several mechanisms are involved in the development of AF in the context of acute circulatory failure, including hypovolemia and β1-adrenergic stimulation in response to endogenous catecholamine production as well as norepinephrine infusion [3]. Atrial fibrillation impairs left ventricular filling and consecutively stroke volume, and in fine potentially aggravates circulatory failure.

Pharmacological options to control AF-related tachycardia are limited. Calcium channel blockers are not frequently used because of long-term negative inotropic effects. Amiodarone is the most used drug but its optimal dosage to fine tune heart rate remains an issue, as well as its potential lung toxicity, especially in case of acute respiratory disease. Landiolol is a beta-blocker with highly β1 selective activity, used either in AF patients either to control heart rate or to prevent supraventricular arrhythmia occurrence in the context of cardiac surgery. Landiolol has an ultrashort half-life of 4 min and weaker negative inotropic effect compared with other intravenous β-blockers [4]. A recent randomized controlled trial in patient with sepsis/septic shock developing tachyarrhythmia showed that Landiolol infusion efficiently reduced heart rate without any significant hemodynamic side effect [5].

Here, we described in critically ill patients admitted to the ICU for SARS-CoV-2 infections presenting with AF, our experience of Landiolol use in terms of efficacy and safety.

## Methods

In our 18-bed intensive care unit, we prospectively collected data from adult patients admitted for SARS-CoV-2 infections with persistent AF. When heart rate was over 120 bpm, Landiolol was started at a minimum dose of 0.2 µg/kg/min and progressively increased by steps of 0.2–0.4 µg/kg/min every hour to achieve 20% reduction in heart rate, without bolus. Hemodynamic parameters were recorded every 2 h during the first 24 h of drug infusion.

## Results

**Fig. 1:**
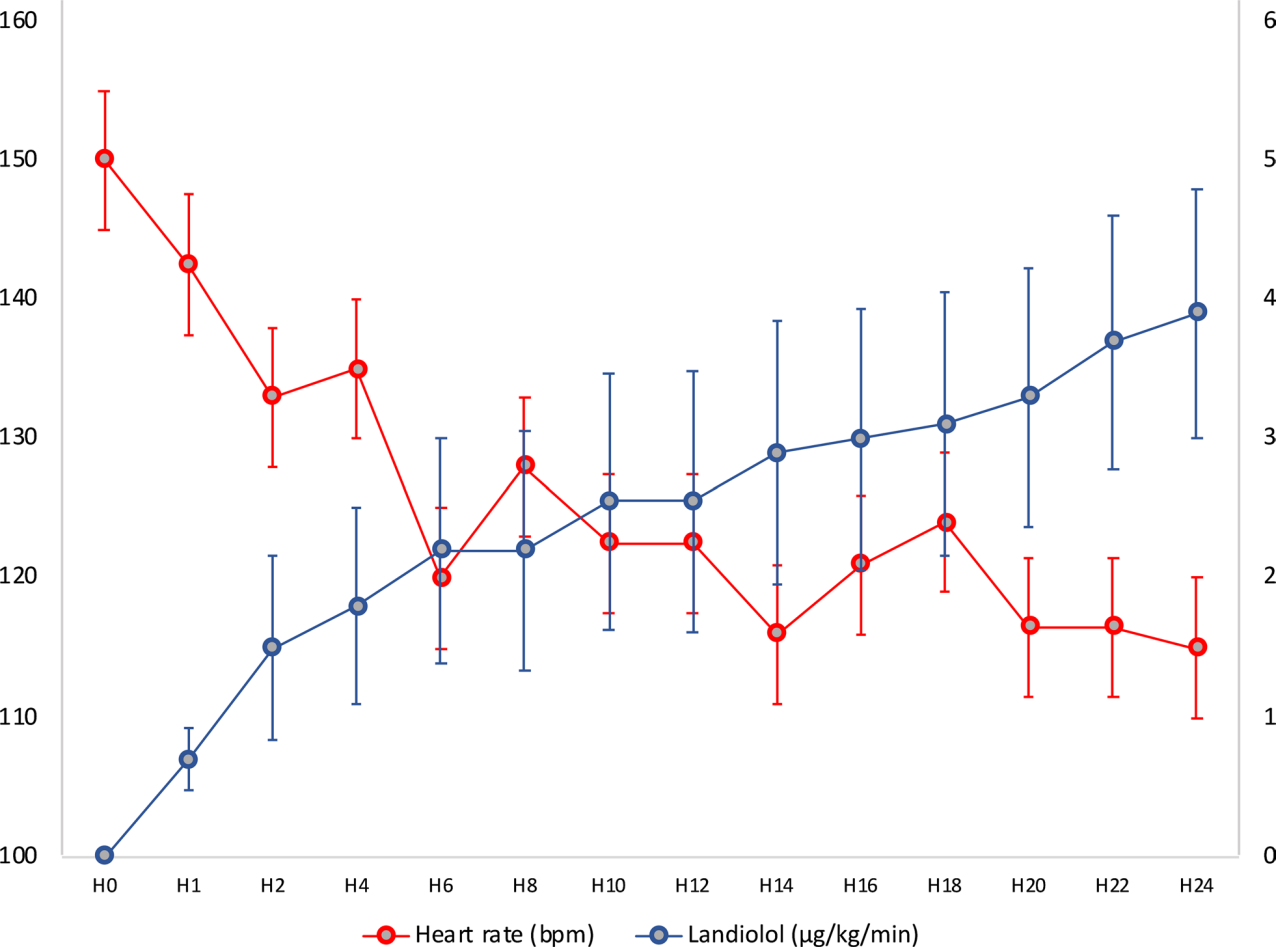
Heart rate (Red) and Landiolol dosage (blue) changes during the first 24 h of Landiolol infusion

Fifteen consecutive patients with SARS-CoV-2 infection were treated with Landiolol during a 6-month period. Median age was 70 [67–72] years old, 27% were female, and median SOFA score was 11 [7–12]. Six patients (6/15, 40%) had a history of chronic AF, and the other had recent AF resistant to electric cardioversion. Median left ventricular (LV) function was 55% [50–57] (Table 1) and no patient had LV ejection fraction < 40%. All included patients underwent invasive mechanical ventilation support and eleven (11/15, 73%) required norepinephrine. Median time between ICU admission and Landiolol initiation was 2 [0–5] days. Landiolol infusion was started at 0.2 µg/kg/min and dosage reached 3.9 [1.6–7.0] µg/kg/min at 24 h. Overall heart rate reduction was 23% (115 [108–117] vs 150 [138–160] bpm; *p* < 0.01, Wilcoxon signed-rank test), without any negative impact on global hemodynamic or tissue perfusion parameters (Table 1). Interestingly, during Landiolol infusion, norepinephrine need decreased in 9/11 patients (81%), and mean norepinephrine dose significantly decreased (0.7 [0.2–1] vs 1 [0.4–1.5] µg/kg/min; *p* = 0.04, Wilcoxon signed-rank test) (Fig. 1).

**Table 1:** Global hemodynamic and tissue perfusion parameters before and after (H24) Landiolol initiation

Patient characteristics	*n* = 15
Age (years old)	70 [67–72]
Women, *n* (%)	4 (27)
SOFA	11 [7–12]
Left ventricular ejection fraction (LVEF) (%)	55 [50–57]
LVEF ≥ 60% (*n*)	4/15
LVEF ≥ 50% and < 60% (*n*)	8/15
LVEF ≥ 40% and < 50% (*n*)	3/15
Time between ICU admission and injection (days)	2 [0–5]
Comorbidities, *n* (%)	
Arterial hypertension	8 (53)
Diabetes mellitus	4 (27)
Vascular disease	5 (33)
Norepinephrine, *n* (%)	11 (73)
Mechanical ventilation, *n* (%)	15 (100)

Hemodynamic kinetics	H0	H24	*p*
Global hemodynamics			
Heart rate (bpm)	150 [138–160]	115 [108–117]	< 0.01
Systolic blood pressure (mmHg)	115 [99–130]	120 [108–125]	0.70
Diastolic blood pressure (mmHg)	58 [51–74]	60 [53–66]	0.71
Mean arterial pressure (mmHg)	79 [69–89]	76 [72–88]	0.76
Norepinephrine dose (µg/kg/min)	1 [0.4–1.5]	0.7 [0.2–1]	0.04
Tissue perfusion parameters			
Index capillary refill time (s)	2 [1.5–2.5]	2 [1.6–2.5]	0.75
Mottling score *n* (%)			0.62
0–1	12 (80)	13 (87)	
2–3	1 (7)	2 (13)	
> 4	2 (13)	0 (0)	
Arterial lactate level (mmol/L)	3.2 [2–4.9]	2.2 [1.7–2.8]	0.05

## Discussion

In this observational uncontrolled study, Landiolol was safely used to lower heart rate in critically ill patients with AF with normal or moderately altered cardiac function. Given that most patients under mechanical ventilation for SARS-CoV-2 infections received norepinephrine and that some of them had moderate left ventricular systolic function, Landiolol infusion was started at very low doses and maximal infusion rate at 24 h was lower than reported in studies on non-critically ill patients [6]. Using this protocol, hemodynamic tolerance was excellent without any significant arterial hypotension or alteration in peripheral tissue perfusion. Moreover, we observed a decrease in norepinephrine need after Landiolol initiation. Additional studies are required to investigate the potential beneficial mechanisms of Landiolol on global hemodynamics.

## Data Availability

The datasets used and/or analyzed during the current study are available from the corresponding author on reasonable request.
